# *N*′-[(*E*)-(3-Fluoro­pyridin-2-yl)methyl­idene]pyridine-3-carbohydrazide dihydrate

**DOI:** 10.1107/S1600536814006072

**Published:** 2014-03-26

**Authors:** Yamuna Nair, M. Sithambaresan, S. Muraleedharan Nair, M. R. Prathapachandra Kurup

**Affiliations:** a Department of Chemical Ocenography, Cochin University of Science and Technology, Lakeside Campus, Kochi 682 016, India; b Department of Chemistry, Faculty of Science, Eastern University, Sri Lanka, Chenkalady, Sri Lanka; c Department of Applied Chemistry, Cochin University of Science and Technology, Kochi 682 022, India

## Abstract

The organic molecule in the title dihydrate, C_12_H_9_FN_4_O·2H_2_O, exists in the *E* conformation with respect to the azo­methane C=N double bond. The mol­ecule is approximately planar, with a maximum deviation of 0.117 (1) Å for the carbonyl O atom from the mean plane of the mol­ecule. Both pyridine rings are essentially coplanar with the central C(=O)N_2_C unit [dihedral angles = 1.99 (7) and 5.71 (8)°], exhibiting a significant difference in dihedral angles from its benzohydrazide analogue. The crystal packing features N—H⋯O, O—H⋯N and O—H⋯O hydrogen-bond inter­actions, which lead to the formation of a chain along the *c-*axis direction through one of the water mol­ecules present, and these chains are stacked one over the other by means of π–π inter­actions [with centroid–centroid distances of 3.7099 (10) and 3.6322 (10) Å] between the aromatic rings in neighbouring anti­parallel mol­ecules, building a three-dimensional supra­molecular network.

## Related literature   

For the biological activity of carbohydrazide derivatives, see: Sreeja *et al.* (2004 ▶); Havanur *et al.* (2010 ▶); Despaigne *et al.* (2010 ▶). For the synthesis of related compounds, see: Kuriakose *et al.* (2007 ▶). For a related structure, see Nair *et al.* (2012 ▶).
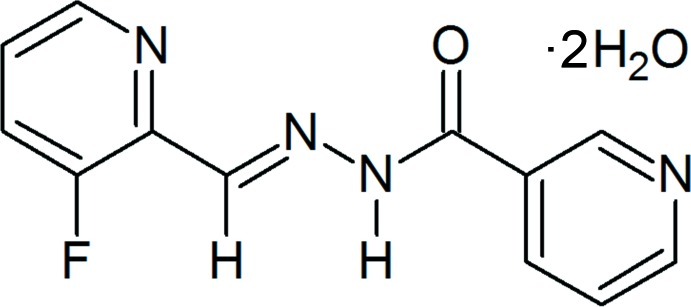


## Experimental   

### 

#### Crystal data  

C_12_H_9_FN_4_O·2H_2_O
*M* *_r_* = 280.26
Monoclinic, 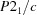

*a* = 7.3023 (7) Å
*b* = 14.4031 (17) Å
*c* = 12.6422 (13) Å
β = 94.842 (3)°
*V* = 1324.9 (2) Å^3^
*Z* = 4
Mo *K*α radiation
μ = 0.11 mm^−1^
*T* = 296 K
0.41 × 0.21 × 0.20 mm

#### Data collection  

Bruker Kappa APEXII CCD diffractometer
Absorption correction: multi-scan (*SADABS*; Bruker, 2004 ▶) *T*_min_ = 0.963, *T*_max_ = 0.969
9779 measured reflections
3237 independent reflections
2339 reflections with *I* > 2σ(*I*)
*R*_int_ = 0.030

#### Refinement  

*R*[*F* ^2^ > 2σ(*F* ^2^)] = 0.048
*wR*(*F* ^2^) = 0.149
*S* = 1.04
3237 reflections
202 parameters
7 restraints
H atoms treated by a mixture of independent and constrained refinement
Δρ_max_ = 0.29 e Å^−3^
Δρ_min_ = −0.20 e Å^−3^

### 

Data collection: *APEX2* (Bruker, 2004 ▶); cell refinement: *APEX2* and *SAINT* (Bruker, 2004 ▶); data reduction: *SAINT* and *XPREP* (Bruker, 2004 ▶); program(s) used to solve structure: *SHELXS97* (Sheldrick, 2008 ▶); program(s) used to refine structure: *SHELXL97* (Sheldrick, 2008 ▶); molecular graphics: *ORTEP-3 for Windows* (Farrugia, 2012 ▶) and *DIAMOND* (Brandenburg, 2010 ▶); software used to prepare material for publication: *SHELXL97* and *publCIF* (Westrip, 2010 ▶).

## Supplementary Material

Crystal structure: contains datablock(s) Global, I. DOI: 10.1107/S1600536814006072/bv2232sup1.cif

Structure factors: contains datablock(s) I. DOI: 10.1107/S1600536814006072/bv2232Isup2.hkl

Supporting information file. DOI: 10.1107/S1600536814006072/bv2232Isup3.cml

CCDC reference: 992525

Additional supporting information:  crystallographic information; 3D view; checkCIF report

## Figures and Tables

**Table 1:** Hydrogen-bond geometry (Å, °)

*D*—H⋯*A*	*D*—H	H⋯*A*	*D*⋯*A*	*D*—H⋯*A*
N3—H3′⋯O1*S*	0.88 (1)	2.04 (1)	2.8821 (19)	160 (2)
O1*S*—H1*A*⋯N4^i^	0.87 (1)	2.09 (1)	2.946 (2)	170 (3)
O2*S*—H2*A*⋯N1^i^	0.87 (1)	2.10 (1)	2.965 (2)	177 (2)
O2*S*—H2*B*⋯O1^i^	0.86 (1)	1.97 (1)	2.816 (2)	172 (2)

Symmetry code: (i) 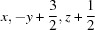.

**Fig. 1.**
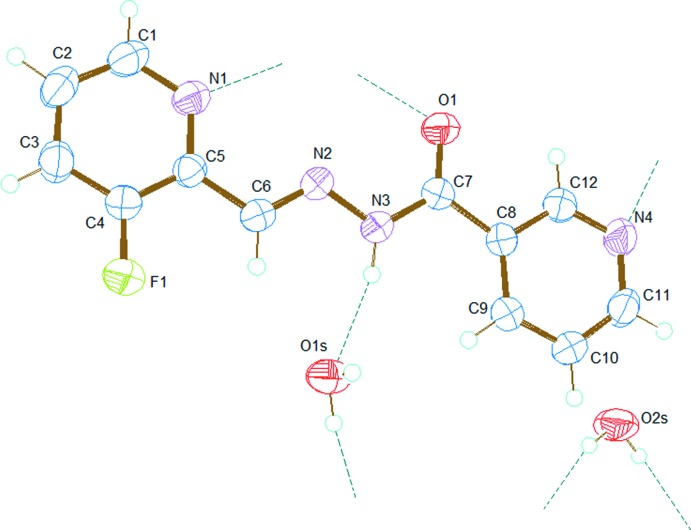
ORTEP diagram of N'-[(E)-(3-fluoropyridin-2-yl)methylidene]pyridine-3-carbohydrazide dihydrate with 50% probability ellipsoids.

**Fig. 2.**
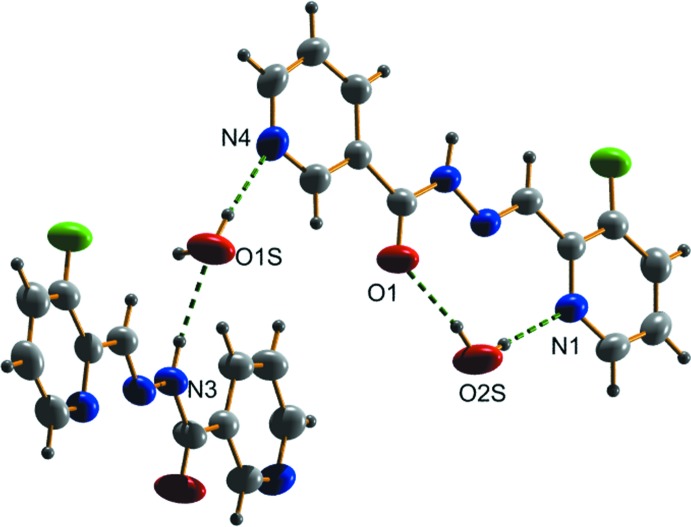
Hydrogen-bonding interactions showing the interconnection of the molecules via one of the water molecules in the lattice.

**Fig. 3.**
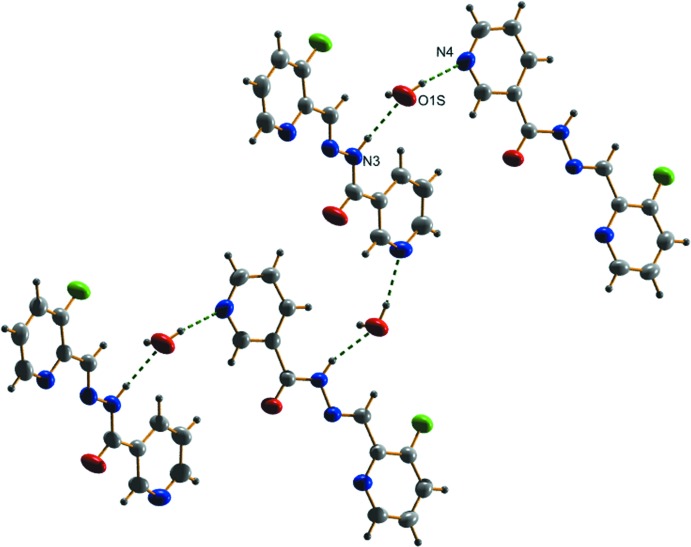
Hydrogen-bonding interactions showing the chain progressing along c axis.

**Fig. 4.**
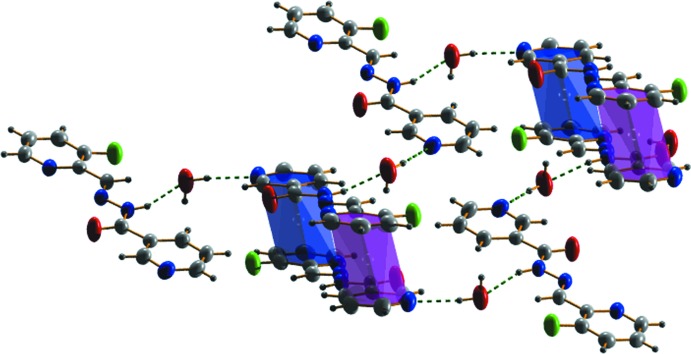
Hydrogen-bonding and π–π interactions in the lattice.

**Fig. 5.**
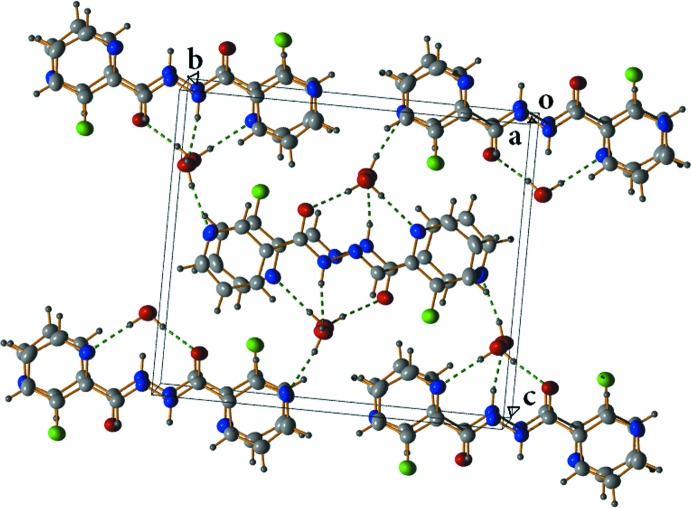
Packing diagram showing the stacked packing arrangement of the molecules along a axis.

## supplementary crystallographic information

### 1. Comment

Carbohydrazides have attracted much attention for their excellent biological properties. Moreover, carbohydrazides derived from 2-acetylpyridine are known to inhibit the proliferation of tumour cells to a greater extent compared to standard anticancer agents (Havanur *et al.*, 2010; Sreeja *et al.*, 2004). In addition, metal complexes with carbohydrazides exhibit antimicrobial, DNA-binding and cytotoxic activities. It has also been shown that these metal complexes can be potent inhibitors of cell growth and DNA synthesis (Despaigne *et al.*, 2010). We report herein the crystal structure of the title compound, a new carbohydrazide.

This molecule adopts an *E* configuration (Fig. 1) with respect to the C6=N2 bond and it exists in the amido form with a C7=O1 bond length of 1.2211 (18) Å which is very close to the reported C=O bond length of similar structure of benzene analogue (Nair *et al.*, 2012). The O1 and N2 atoms are in a *Z* configuration with respect to C7–N3 having a tortional angle of -0.6 (3)°. The molecule is almost planar with maximum deviation of 0.117 (1) Å for the atom O1 from the mean plane of the molecule (r.m.s. deviation, 0.0513). The pyridyl ring having F atom is essentially coplanar with the central C(=O)N_2_C unit (dihedral angle 5.71 (8)°), the other pyridyl ring exhibits a torsion angle of 1.99 (7)°.

Whilst one of the water molecules connects two adjacent molecules through two O–H···N and N–H···O H-bonding interactions with D···A distances of 2.946 (2) and 2.882 (1) Å respectively, the other water molecule forms two O–H···N and O–H···O H-bonds with D···A distances of 2.965 (2) and 2.816 (2) Å with the same molecule (Fig. 2, Table 1). One of the water molecules acts as both a hydrogen bond acceptor as well as a donor towards another carbohydrazide molecule while the other acts only as hydrogen bond donor. By means of these interactions the molecules are chained through one of the water molecules to form infinite chains parallel to the *c* axis of the unit cell (Fig. 3). These parallel chains are stacked one over the other by means of two π–π interactions between the two aromatic rings of the neighbouring anti parallel molecules (Fig. 4) with centeroid-centeroid distances of 3.7099 (10) and 3.6322 (10) Å. Fig. 5 shows the stacked packing of the molecules along *a* axis in the unit cell.

### 2. Experimental

The title compound was prepared by adapting a reported procedure (Kuriakose *et al.*, 2007). A solution of 3-fluoropyridine-2-carbaldehyde (1.25 g,1 mmol) in ethanol (10 ml) was mixed with an ethanolic solution (10 ml) of pyridine-3-carbohydrazide (1.37 g,1 mmol). The mixture was boiled under reflux for 12 h after adding few drops of glacial acetic acid and then cooled to room temperature. Colorless needle shaped crystals, suitable for single-crystal analysis, were obtained after slow evaporation of the solution in air for a few days.

### 3. Refinement

The atoms H3', H1A, H1B, H2A and H2B were located from a difference Fourier map and refined isotropically. The N3—H3' bond distance was restrained to 0.88±0.01 Å. The O—H distances of water were restrained to 0.86±0.01 Å and H···H distances to 1.36±0.02 Å. The remaining hydrogen atoms were placed in geometrically idealized positions and constrained to ride on their parent atoms, with C–H distances of 0.93 Å, and with isotropic displacement parameters 1.2 times that of the parent carbon atoms.

### Crystal data

**Table:**

C_12_H_9_FN_4_O·2H_2_O	*F*(000) = 584
*M**_r_* = 280.26	*D*_x_ = 1.405 Mg m^−^^3^
Monoclinic, *P*2_1_/*c*	Mo *K*α radiation, λ = 0.71073 Å
Hall symbol: -P 2ybc	Cell parameters from 4225 reflections
*a* = 7.3023 (7) Å	θ = 2.8–28.0°
*b* = 14.4031 (17) Å	µ = 0.11 mm^−^^1^
*c* = 12.6422 (13) Å	*T* = 296 K
β = 94.842 (3)°	Needle, colorless
*V* = 1324.9 (2) Å^3^	0.41 × 0.21 × 0.20 mm
*Z* = 4	

### Data collection

**Table:**

Bruker Kappa APEXII CCD diffractometer	3237 independent reflections
Radiation source: fine-focus sealed tube	2339 reflections with *I* > 2σ(*I*)
Graphite monochromator	*R*_int_ = 0.030
Detector resolution: 8.33 pixels mm^-1^	θ_max_ = 28.2°, θ_min_ = 2.8°
ω and φ scan	*h* = −9→9
Absorption correction: multi-scan (*SADABS*; Bruker, 2004)	*k* = −17→19
*T*_min_ = 0.963, *T*_max_ = 0.969	*l* = −16→16
9779 measured reflections	

### Refinement

**Table:**

Refinement on *F*^2^	Secondary atom site location: difference Fourier map
Least-squares matrix: full	Hydrogen site location: inferred from neighbouring sites
*R*[*F*^2^ > 2σ(*F*^2^)] = 0.048	H atoms treated by a mixture of independent and constrained refinement
*wR*(*F*^2^) = 0.149	*w* = 1/[σ^2^(*F*_o_^2^) + (0.0712*P*)^2^ + 0.3471*P*] where *P* = (*F*_o_^2^ + 2*F*_c_^2^)/3
*S* = 1.04	(Δ/σ)_max_ < 0.001
3237 reflections	Δρ_max_ = 0.29 e Å^−^^3^
202 parameters	Δρ_min_ = −0.20 e Å^−^^3^
7 restraints	Extinction correction: *SHELXL97* (Sheldrick, 2008), Fc^*^=kFc[1+0.001xFc^2^λ^3^/sin(2θ)]^-1/4^
Primary atom site location: structure-invariant direct methods	Extinction coefficient: 0.079 (6)

### Special details

**Table:**

Geometry. All e.s.d.'s (except the e.s.d. in the dihedral angle between two l.s. planes) are estimated using the full covariance matrix. The cell e.s.d.'s are taken into account individually in the estimation of e.s.d.'s in distances, angles and torsion angles; correlations between e.s.d.'s in cell parameters are only used when they are defined by crystal symmetry. An approximate (isotropic) treatment of cell e.s.d.'s is used for estimating e.s.d.'s involving l.s. planes.
Refinement. Refinement of *F*^2^ against ALL reflections. The weighted *R*-factor *wR* and goodness of fit *S* are based on *F*^2^, conventional *R*-factors *R* are based on *F*, with *F* set to zero for negative *F*^2^. The threshold expression of *F*^2^ > σ(*F*^2^) is used only for calculating *R*-factors(gt) *etc*. and is not relevant to the choice of reflections for refinement. *R*-factors based on *F*^2^ are statistically about twice as large as those based on *F*, and *R*- factors based on ALL data will be even larger.

### Fractional atomic coordinates and isotropic or equivalent isotropic displacement parameters (Å^2^)

**Table:**

	*x*	*y*	*z*	*U*_iso_*/*U*_eq_	
O2S	0.5863 (3)	0.45901 (12)	0.25212 (11)	0.0835 (5)	
F1	0.77433 (17)	1.26247 (8)	0.16447 (8)	0.0683 (4)	
O1	0.7242 (2)	0.88186 (10)	−0.13894 (9)	0.0770 (5)	
O1S	0.7875 (3)	0.96417 (11)	0.24957 (11)	0.0792 (5)	
N1	0.60794 (18)	1.20176 (10)	−0.10083 (10)	0.0470 (3)	
C6	0.7337 (2)	1.10213 (11)	0.04205 (11)	0.0427 (4)	
H6	0.7710	1.0945	0.1137	0.051*	
N2	0.73006 (18)	1.03335 (9)	−0.02053 (10)	0.0440 (3)	
N4	0.8628 (2)	0.62144 (10)	−0.03962 (11)	0.0543 (4)	
C1	0.5563 (2)	1.28602 (13)	−0.13628 (13)	0.0530 (4)	
H1	0.5071	1.2915	−0.2063	0.064*	
C2	0.5718 (2)	1.36560 (12)	−0.07482 (15)	0.0548 (4)	
H2	0.5331	1.4226	−0.1031	0.066*	
C3	0.6450 (3)	1.35901 (12)	0.02835 (14)	0.0540 (4)	
H3	0.6583	1.4109	0.0721	0.065*	
C4	0.6980 (2)	1.27194 (11)	0.06413 (12)	0.0455 (4)	
C5	0.6785 (2)	1.19411 (10)	0.00027 (11)	0.0396 (3)	
N3	0.78095 (19)	0.94872 (9)	0.02199 (10)	0.0435 (3)	
C7	0.7743 (2)	0.87503 (11)	−0.04464 (11)	0.0452 (4)	
C8	0.8311 (2)	0.78321 (10)	0.00146 (11)	0.0396 (3)	
C9	0.8966 (2)	0.76657 (11)	0.10573 (12)	0.0475 (4)	
H9	0.9101	0.8151	0.1543	0.057*	
C10	0.9414 (2)	0.67696 (12)	0.13635 (13)	0.0514 (4)	
H10	0.9836	0.6638	0.2062	0.062*	
C11	0.9223 (2)	0.60731 (12)	0.06144 (14)	0.0530 (4)	
H11	0.9528	0.5471	0.0828	0.064*	
C12	0.8172 (2)	0.70823 (12)	−0.06745 (12)	0.0479 (4)	
H12	0.7734	0.7191	−0.1376	0.057*	
H3'	0.809 (3)	0.9462 (13)	0.0910 (8)	0.057 (5)*	
H2A	0.597 (3)	0.4119 (11)	0.2949 (16)	0.088 (8)*	
H1A	0.807 (4)	0.9453 (19)	0.3148 (10)	0.110 (9)*	
H2B	0.623 (4)	0.5053 (11)	0.2904 (17)	0.097 (9)*	
H1B	0.902 (2)	0.962 (3)	0.233 (3)	0.19 (2)*	

### Atomic displacement parameters (Å^2^)

**Table:**

	*U*^11^	*U*^22^	*U*^33^	*U*^12^	*U*^13^	*U*^23^
O2S	0.1384 (15)	0.0589 (9)	0.0478 (7)	−0.0012 (9)	−0.0228 (8)	−0.0033 (7)
F1	0.1063 (9)	0.0553 (6)	0.0417 (5)	0.0069 (6)	−0.0038 (5)	−0.0027 (4)
O1	0.1404 (14)	0.0510 (8)	0.0373 (6)	0.0225 (8)	−0.0057 (7)	−0.0003 (5)
O1S	0.1276 (15)	0.0674 (9)	0.0409 (7)	0.0091 (9)	−0.0028 (8)	0.0039 (6)
N1	0.0501 (7)	0.0474 (8)	0.0426 (7)	0.0026 (6)	−0.0009 (5)	0.0049 (6)
C6	0.0482 (8)	0.0418 (8)	0.0377 (7)	0.0021 (6)	0.0007 (6)	0.0040 (6)
N2	0.0547 (8)	0.0379 (7)	0.0393 (6)	0.0069 (5)	0.0033 (5)	0.0047 (5)
N4	0.0634 (9)	0.0430 (8)	0.0545 (8)	0.0091 (6)	−0.0071 (6)	−0.0092 (6)
C1	0.0532 (10)	0.0578 (10)	0.0474 (8)	0.0067 (8)	0.0002 (7)	0.0133 (7)
C2	0.0565 (10)	0.0453 (9)	0.0638 (10)	0.0106 (7)	0.0123 (8)	0.0164 (8)
C3	0.0659 (11)	0.0395 (9)	0.0584 (10)	0.0046 (7)	0.0159 (8)	0.0007 (7)
C4	0.0525 (9)	0.0449 (8)	0.0397 (7)	0.0033 (7)	0.0069 (6)	0.0023 (6)
C5	0.0403 (8)	0.0389 (8)	0.0400 (7)	0.0023 (6)	0.0061 (6)	0.0042 (6)
N3	0.0568 (8)	0.0372 (7)	0.0360 (6)	0.0067 (5)	0.0011 (5)	0.0033 (5)
C7	0.0582 (9)	0.0418 (8)	0.0359 (7)	0.0072 (7)	0.0060 (6)	0.0005 (6)
C8	0.0404 (8)	0.0397 (8)	0.0390 (7)	0.0041 (6)	0.0048 (6)	−0.0008 (6)
C9	0.0596 (10)	0.0420 (8)	0.0406 (8)	0.0068 (7)	0.0021 (7)	−0.0035 (6)
C10	0.0610 (10)	0.0500 (9)	0.0419 (8)	0.0113 (7)	−0.0033 (7)	0.0036 (7)
C11	0.0577 (10)	0.0408 (9)	0.0589 (10)	0.0112 (7)	−0.0036 (8)	0.0016 (7)
C12	0.0540 (9)	0.0465 (9)	0.0417 (8)	0.0070 (7)	−0.0041 (6)	−0.0045 (6)

### Geometric parameters (Å, º)

**Table:**

O2S—H2A	0.866 (9)	C2—C3	1.371 (2)
O2S—H2B	0.855 (9)	C2—H2	0.9300
F1—C4	1.3486 (18)	C3—C4	1.377 (2)
O1—C7	1.2211 (18)	C3—H3	0.9300
O1S—H1A	0.869 (10)	C4—C5	1.382 (2)
O1S—H1B	0.881 (10)	N3—C7	1.3534 (19)
N1—C1	1.337 (2)	N3—H3'	0.880 (9)
N1—C5	1.3417 (18)	C7—C8	1.490 (2)
C6—N2	1.267 (2)	C8—C9	1.385 (2)
C6—C5	1.470 (2)	C8—C12	1.386 (2)
C6—H6	0.9300	C9—C10	1.379 (2)
N2—N3	1.3707 (17)	C9—H9	0.9300
N4—C11	1.330 (2)	C10—C11	1.379 (2)
N4—C12	1.333 (2)	C10—H10	0.9300
C1—C2	1.384 (3)	C11—H11	0.9300
C1—H1	0.9300	C12—H12	0.9300
			
H2A—O2S—H2B	104.5 (17)	C4—C5—C6	120.63 (13)
H1A—O1S—H1B	97.5 (19)	C7—N3—N2	117.32 (12)
C1—N1—C5	117.95 (14)	C7—N3—H3'	125.3 (13)
N2—C6—C5	119.32 (13)	N2—N3—H3'	117.3 (13)
N2—C6—H6	120.3	O1—C7—N3	122.44 (14)
C5—C6—H6	120.3	O1—C7—C8	120.26 (14)
C6—N2—N3	117.37 (13)	N3—C7—C8	117.29 (13)
C11—N4—C12	116.80 (14)	C9—C8—C12	117.69 (14)
N1—C1—C2	123.80 (15)	C9—C8—C7	126.03 (13)
N1—C1—H1	118.1	C12—C8—C7	116.28 (13)
C2—C1—H1	118.1	C10—C9—C8	118.94 (14)
C3—C2—C1	118.92 (15)	C10—C9—H9	120.5
C3—C2—H2	120.5	C8—C9—H9	120.5
C1—C2—H2	120.5	C11—C10—C9	118.72 (15)
C2—C3—C4	116.87 (16)	C11—C10—H10	120.6
C2—C3—H3	121.6	C9—C10—H10	120.6
C4—C3—H3	121.6	N4—C11—C10	123.68 (15)
F1—C4—C3	118.90 (15)	N4—C11—H11	118.2
F1—C4—C5	118.79 (14)	C10—C11—H11	118.2
C3—C4—C5	122.30 (15)	N4—C12—C8	124.16 (14)
N1—C5—C4	120.16 (14)	N4—C12—H12	117.9
N1—C5—C6	119.21 (13)	C8—C12—H12	117.9
			
C5—C6—N2—N3	−179.37 (13)	N2—N3—C7—O1	−0.6 (3)
C5—N1—C1—C2	0.1 (3)	N2—N3—C7—C8	179.54 (13)
N1—C1—C2—C3	0.4 (3)	O1—C7—C8—C9	177.57 (17)
C1—C2—C3—C4	−0.3 (3)	N3—C7—C8—C9	−2.5 (2)
C2—C3—C4—F1	178.65 (15)	O1—C7—C8—C12	−2.0 (2)
C2—C3—C4—C5	−0.3 (3)	N3—C7—C8—C12	177.88 (14)
C1—N1—C5—C4	−0.8 (2)	C12—C8—C9—C10	−1.2 (2)
C1—N1—C5—C6	179.01 (14)	C7—C8—C9—C10	179.25 (15)
F1—C4—C5—N1	−178.09 (13)	C8—C9—C10—C11	1.1 (3)
C3—C4—C5—N1	0.9 (2)	C12—N4—C11—C10	−1.1 (3)
F1—C4—C5—C6	2.1 (2)	C9—C10—C11—N4	0.0 (3)
C3—C4—C5—C6	−178.89 (15)	C11—N4—C12—C8	1.1 (3)
N2—C6—C5—N1	6.6 (2)	C9—C8—C12—N4	0.0 (3)
N2—C6—C5—C4	−173.62 (15)	C7—C8—C12—N4	179.68 (16)
C6—N2—N3—C7	179.03 (14)		

### Hydrogen-bond geometry (Å, º)

**Table:**

*D*—H···*A*	*D*—H	H···*A*	*D*···*A*	*D*—H···*A*
N3—H3′···O1*S*	0.88 (1)	2.04 (1)	2.8821 (19)	160 (2)
O2*S*—H2*A*···N1^i^	0.87 (1)	2.10 (1)	2.965 (2)	177 (2)
O1*S*—H1*A*···N4^i^	0.87 (1)	2.09 (1)	2.946 (2)	170 (3)
O2*S*—H2*B*···O1^i^	0.86 (1)	1.97 (1)	2.816 (2)	172 (2)

Symmetry code: (i) *x*, −*y*+3/2, *z*+1/2.
